# Long-read Sequencing Data Reveals Dynamic Evolution of Mitochondrial Genome Size and the Phylogenetic Utility of Mitochondrial DNA in Hercules Beetles (*Dynastes*; Scarabaeidae)

**DOI:** 10.1093/gbe/evac147

**Published:** 2022-09-29

**Authors:** Brett Morgan, Tzi-Yuan Wang, Yi-Zhen Chen, Victor Moctezuma, Oscar Burgos, My Hanh Le, Jen-Pan Huang

**Affiliations:** Biodiversity Research Center, Academia Sinica, Taipei, Taiwan; Smithsonian Environmental Research Center, Edgewater, Maryland; Biodiversity Research Center, Academia Sinica, Taipei, Taiwan; Department of Entomology, National Taiwan University, Taipei, Taiwan; Centro Tlaxcala de Biología de la Conducta, Universidad Autónoma de Tlaxcala, Tlaxcala de Xicohténcatl, Tlaxcala, Mexico; Centro de Investigaciones Biológicas, Universidad Autónoma del Estado de Morelos, Cuernavaca, Mexico; Biodiversity Research Center, Academia Sinica, Taipei, Taiwan; Biodiversity Research Center, Academia Sinica, Taipei, Taiwan

**Keywords:** control region, long-read sequencing, phylogenetic discordance, purifying selection, mtDNA, short-read sequencing

## Abstract

The evolutionary dynamics and phylogenetic utility of mitochondrial genomes (mitogenomes) have been of particular interest to systematists and evolutionary biologists. However, certain mitochondrial features, such as the molecular evolution of the control region in insects, remain poorly explored due to technological constraints. Using a combination of long- and short-read sequencing data, we assembled ten complete mitogenomes from ten Hercules beetles. We found large-sized mitogenomes (from 24 to 28 kb), which are among the largest in insects. The variation in genome size can be attributed to copy-number evolution of tandem repeats in the control region. Furthermore, one type of tandem repeat was found flanking the conserved sequence block in the control region. Importantly, such variation, which made up around 30% of the size of the mitogenome, may only become detectable should long-read sequencing technology be applied. We also found that, although different mitochondrial loci often inferred different phylogenetic histories, none of the mitochondrial loci statistically reject a concatenated mitochondrial phylogeny, supporting the hypothesis that all mitochondrial loci share a single genealogical history. We on the other hand reported statistical support for mito-nuclear phylogenetic discordance in 50% of mitochondrial loci. We argue that long-read DNA sequencing should become a standard application in the rapidly growing field of mitogenome sequencing. Furthermore, mitochondrial gene trees may differ even though they share a common genealogical history, and *ND* loci could be better candidates for phylogenetics than the commonly used *COX1*.

**Significance**

Mitochondrial DNA has been the most commonly used molecular data type to study animal evolution and diversity. Using long-read sequencing data, we discovered two types of tandem repeats in the control region of the mitogenome of Hercules beetles; additionally, the mitogenome sizes are among the largest in insects. When only short-read sequencing data were used, however, we failed to assemble the mitogenomes properly due to limitations of short sequence length. We further showed that *ND5* is a better candidate than *COX1* for molecular phylogenetics in our study system. In conclusion, novel, or previously neglected, properties can be discovered in a well-studied system, such as the animal mitogenome, when new technologies become available and thorough analyses are applied.

## Introduction

Mitochondrial DNA (mtDNA) has been and will continue to be the study focus in animal molecular systematics and evolution (Desalle et al. 2017). Because of its relative ease to be sequenced and a higher mutation rate compared with nuclear loci, the use of mtDNA formed the foundation of phylogeography and revolutionized species identification and delineation (Avise et al. 1987; Zhang and Hewitt 1997; Hebert et al. 2003; Rubinoff and Holland 2005; Pons et al. 2006; Cameron 2014; Desalle et al. 2017). Even in the era of phylogenomics, where large-scale nuclear-genomic data are used to infer evolutionary history, mtDNA still proves to be helpful (e.g., Wu et al. 2020). Additionally, the advances in next-generation sequencing technologies have not only greatly reduced sequencing cost, but have also resulted in the rapid accumulation of mtDNA data either as targets or by-products from various studies (e.g., Raposo do Amaral 2015; Yoshizawa et al. 2018; Tang et al. 2019). The influx of newly available data has been instrumental in estimating species diversity and evolutionary histories of insufficiently studied organismal lineages (Raposo do Amaral 2015; Tang et al. 2019) and understanding the evolution of the mitochondrial genome (mitogenome) itself (Yoshizawa et al. 2018; Formenti et al. 2021).

In animal systems, it has often been assumed that the gene arrangement and genome size of mitogenomes remain more or less constant across lineages (Boore 1999; Gissi et al. 2008; Cameron 2014; Ladoukakis and Zourus 2017). However, such assumptions have been challenging to test due to the limitations of sequencing technologies. Specifically, many recent mitogenome studies relied exclusively on short-read DNA sequence data and de novo assembly, where the published mitogenomes may have been incomplete because of the difficulties in assembling certain genomic regions. For example, the mitochondrial control region may contain complex sequence structure and tandem repeats in many animal lineages (Bronstein et al. 2018; Filipović et al. 2021; Formenti et al. 2021; Kinkar et al. 2021). Furthermore, the genome size and arrangement can also evolve rapidly (Lavrov and Pett 2016; Chen et al. 2018; Filipović et al. 2021). For example, rapid evolution in tandem repeats has been reported in bark weevils that resulted in a mitogenome of unusually large size (Boyce et al. 1989). A recent study unraveled an excessive accumulation of tandem repeats in the mitogenome of a cnidarian species, which resulted in a circular mitogenome size >90 kb (Novosolov et al. 2022). The evolutionary insights from mitogenome evolution might go undetected should one only apply a short-read sequencing approach, which has been extensively applied in mitogenome assemblies in recent years (Desalle et al. 2017; Filipović et al. 2021; Formenti et al. 2021). With recent advances in long-read sequencing technology, we may finally better investigate the evolution of the gene order and genome size of mitogenomes (De Vivo et al. 2022; Novosolov et al. 2022).

Furthermore, although mito-nuclear phylogenetic discordance has frequently been reported (Shaw 2002; Funk and Omland 2003; Rubinoff and Holland 2005; Toews and Brelsford 2012; Yan et al. 2019; Piccinini et al. 2021), the gene trees of all mitochondrial loci have often been assumed to represent the same genealogical history (Boore 1999; Ladoukakis and Zourus 2017). It remains unsettled whether the mito-nuclear phylogenetic discordance was driven by only a small subset or the majority of the mitochondrial loci. Additionally, there has been an increasing reliance on the *COX1* gene for phylogeography, species identification, species delimitation, and phylogenetic inference (the locus has been abbreviated as *Co1*, *COI*, *cox1*, etc.; Rubinoff and Holland 2005, Pons et al. 2006; Cameron 2014), while the efficacy of other mitochondrial loci on similar study foci has become less explored (Luo et al. 2011). It has been shown that phylogenetic inference can be dependent on which mitochondrial loci were utilized, because loci may differ in the number of variable sites and selection pressure (Zhang et al. 2021). However, how statistically different the gene trees are and whether the statistical differences are correlated with the strength of purifying selection or the number of phylogenetically informative sites remain largely unexplored.

**Fig. 1.**
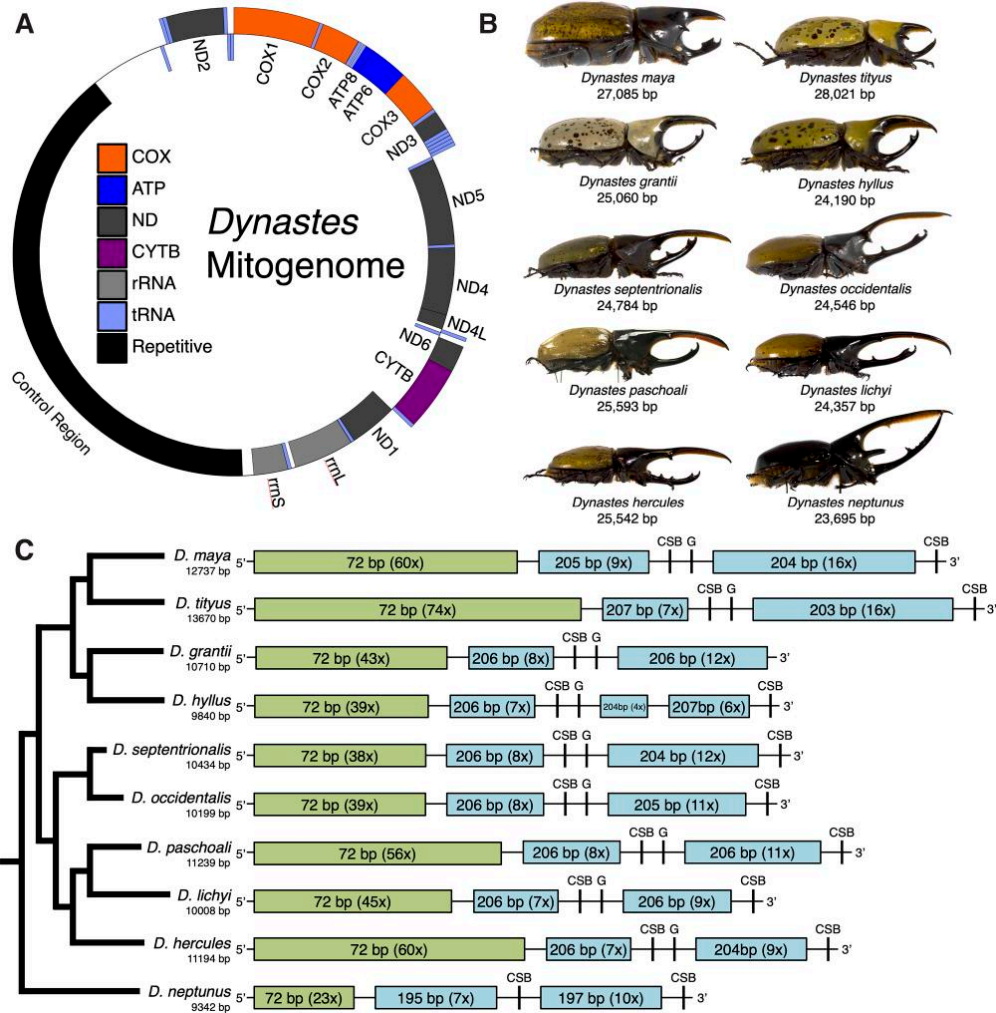
The *Dynastes* beetle mitogenomes. The gene arrangement (*A*) and size (*B*) of the ten newly sequenced and assembled *Dynastes* beetle mitogenomes. Note that the size of the adult male beetles in the images is not scaled. (*C*) The control region contains two types of tandem repeats. A species tree showing the evolutionary relationships among the studied *Dynastes* beetles (Huang 2016a, 2017) is shown to the left of panel *C*. The conserved region in the control region is flanked by the 206-bp tandem repeats in the *Dynastes* mitogenomes. CBS, conserved sequence block; G, poly-G element.

Here we studied the evolution and phylogenetic utility of mtDNA using Hercules beetles (*Dynastes* MacLeay, 1819) as an example, with the application of both long- and short-read sequencing technologies. Hercules beetles are large-sized scarab beetles that can be found in forest habitats of North and South America with 17 currently recognized species (or subspecies; Huang and Knowles 2016; Huang 2017). The adult males of Hercules beetles show high intra- and inter-specific phenotypic diversity in body coloration and male horn structure (fig. 1). The exaggerated male horns and diverse phenotypes have made Hercules beetles foci of evolutionary and taxonomic studies; for example, *Dynastes hercules* was among the first species of Scarab beetles named by Linnaeus (Krell et al. 2012). Moreover, different phylogenetic hypotheses have been proposed for Hercules beetles, where phylogenetic discordances have been revealed when applying different molecular markers (cf., Huang and Knowles 2016; Huang 2016a, 2017). Because of this, the Hercules beetle system can be used to address whether discordant phylogenies can result among mitochondrial loci, and how often mitochondrial loci agree or disagree with the nuclear-genome-based species tree.

Furthermore, as mentioned above, sequencing and assembling complete invertebrate mitogenomes has been challenging because the control region contains various tandem repeats that cannot be properly assembled if only short-read sequencing technologies were employed (e.g., Filipović et al. 2021; Kinkar et al. 2021). Specifically, tandem repeats have been frequently reported in the control region of mitogenomes in many animal lineages (Boore 1999; e.g., Wang et al. 2015; Formenti et al. 2021; Kinkar et al. 2021), including insects (Yuan et al. 2015; Sayadi et al. 2017). However, the DNA sequences of the tandem repeats differ between types of tandem repeats within a mitogenome as well as between mitogenomes from different lineages (e.g., Yuan et al. 2015; Formenti et al. 2021), making a comprehensive study of their evolutionary history challenging. It has been shown that several types of tandem repeats exist in the control region of another dynastine beetle, *Oryctes rhinoceros* (Filipović et al. 2021; but see Ayivi et al. 2021). The use of the Hercules beetle system can help us empirically demonstrate how the application of long-read sequencing technology can or cannot improve mitogenome assemblies compared with approaches using only short-read sequencing technology and unravel the evolutionary history of tandem repeats among closely related species.

We chose to study the evolution of the mitogenome itself and the phylogenetic utility of mtDNA simultaneously, not only because these have been the two main aspects of studying animal mtDNA (Desalle et al. 2017), but also because these two aspects are not independent from each other. For example, the DNA sequence and structural evolution of the control region may inform the phylogenetic relationships (Bronstein et al. 2018). Similarly, closely related species may have a similar copy number of the same type of tandem repeats. In this study, we specifically investigated (1) how variable the mitogenome is in terms of size and gene arrangement and what factors may impact this variability, and (2) how different are the gene trees inferred using different mitochondrial loci and what are the factors that may contribute to this difference, for example, the number of variable sites or the strength of selection.

## Results

### Mitogenomes

By using a combination of long-read (Oxford nanopore technology) and short-read (Illumina) data sets, we successfully assembled and characterized ten complete *Dynastes* mitogenomes. We uncovered the sizes of the ten new assemblies, which range from 23 695 bp&&&& (*Dynastes neptunus*) to 28 021 bp (*Dynastes tityus*; fig. 1). The AT contents of the mitogenomes range from 64.6% (*D. tityus*) to 68.2% (*D. neptunus*). There were 13 protein-coding genes, 2 rRNA loci, and 22 tRNA loci found in each of the 10 *Dynastes* mitogenomes. Additionally, all of the newly sequenced and assembled mitogenomes shared the same gene arrangement (fig. 1). It is important to note that the mitogenomes of *Dynastes* beetles are among the largest of the insect mitogenomes reported so far (Cameron 2014; Sayadi et al. 2017; Filipović et al. 2021). The sequence-read coverage estimated from long-read sequencing data (filtered nanopore reads; see Materials and Methods section; table 1) was even across the entire mitogenome (supplementary figs. S1—S10, Supplementary Material online); on the other hand, the sequencing coverage estimated from short-read sequencing data (Illumina sequencing; table 1) revealed sporadic coverage depth in the control region (supplementary figs. S1—S10, Supplementary Material online). When only short-read data sets were used to assemble and annotate the mitogenomes of *Dynastes hyllus*, *Dynastes septentrionalis*, *Dynastes lichyi*, and *D. neptunus*, the resulting incomplete mitogenomes were much more compact, showing a genome size that was similar to most of the published insect data sets (ca., 16 kb; supplementary fig. S11, Supplementary Material online; Cameron 2014).

**Table 1:** Summary of Sequenced Reads for Mitochondrial-genome Assemblies

Species	Nanopore reads (SRA number) [N50]	Nanopore hits [N50]	Illumina reads (SRA number)	Mt genome size (Accession #)
*D. neptunus*	16.4 Gb (SAMN26642996) [11 644]	234 Mb [14 640]	31 Gb (SAMN26642997)	23.7 kb (ON312101)
*D. occidentalis*	3.1 Gb (SAMN26642998) [5 851]	27 Mb [8 778]	34 Gb (SAMN26642999)	24.5 kb (ON312102)
*D. septentrionalis*	10.9 Gb (SAMN26643000) [5 784]	108 Mb [10 367]	25 Gb (SAMN26643001)	24.8 kb (ON312104)
*D. paschoali*	5.7 Gb (SAMN26643002) [4 805] (SAMN26643003) [2 771]	106 Mb [4 265]	35 Gb (SAMN26643004)	25.6 kb (ON312103)
*D. lichyi*	4.5 Gb (SAMN26643005) [5 768]	45 Mb [9 027]	27 Gb (SAMN26643006)	25.4 kb (ON312099)
*D. hercules*	11.6 Gb (SAMN26643007) [2 584]	94 Mb [2 302]	29 Gb (SAMN26643008)	25.5 kb (ON312097)
*D. grantii*	34.8 Gb (SAMN26643009) [2 442] (SAMN26643010) [4 119]	253 Mb [2 614]	28 Gb (SAMN26643011)	25.1 kb (ON312096)
*D. hyllus*	12.0 Gb (SAMN26643012) [2 586] (SAMN26643013) [2 405] (SAMN26643014) [2 144]	173 Mb [2 333]	33 Gb (SAMN26643015)	24.2 kb (ON312098)
*D. maya*	1.8 Gb (SAMN26688269) [11 149]	105 Mb [3 559]	32 Gb (SAMN26643018) (SAMN26643019)	27.1 kb (ON312100)
*D. tityus*	28.0 Gb (SAMN26643020) [5 433] (SAMN26643021) [3 283]	341 Mb [6 295]	21 Gb (SAMN26643022)	28.0 kb (ON312105)

**Fig. 2.**
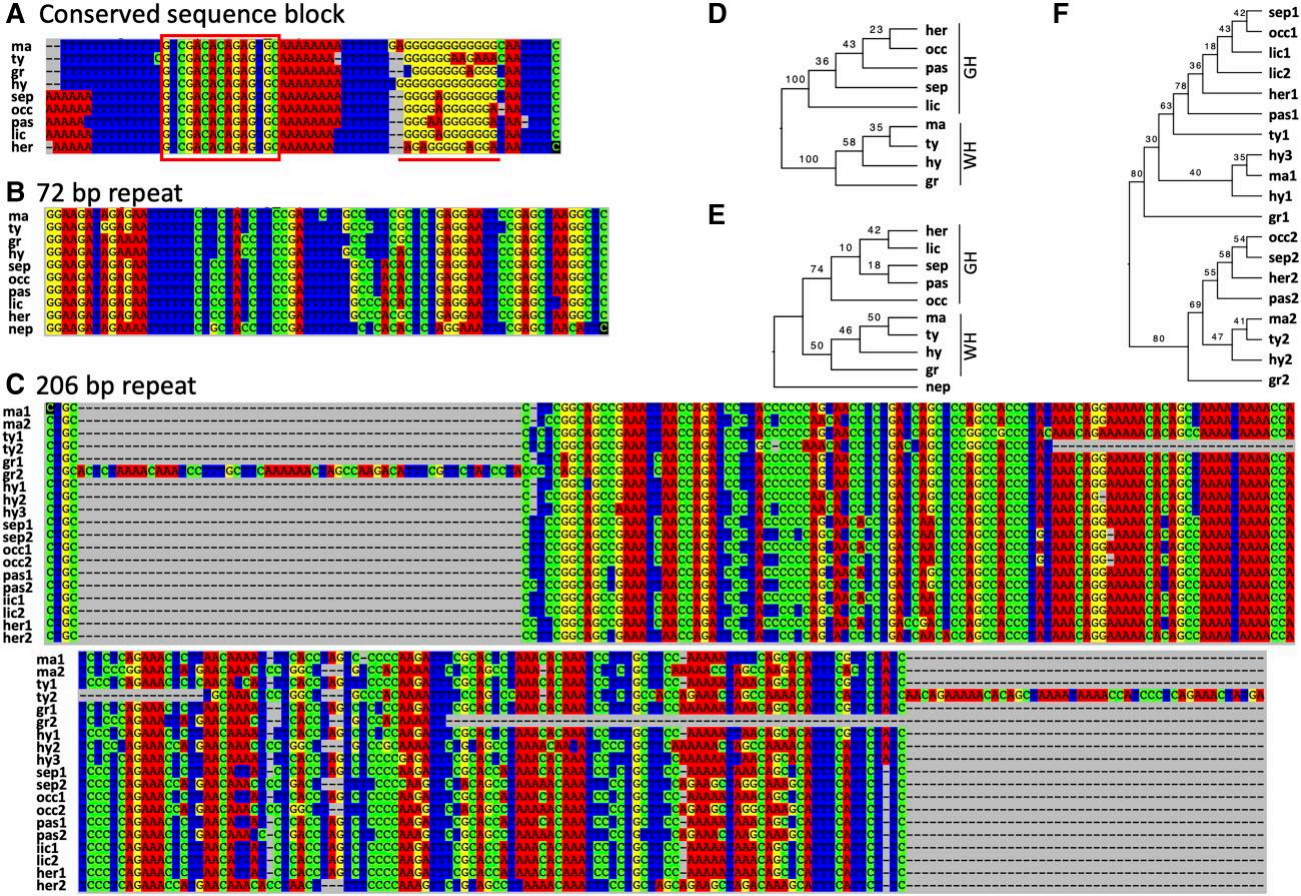
Sequence alignments of the conserved sequence block, 72 bp repeat, 206 bp repeat, and phylogenetic reconstructions based on the sequence alignments. (*A*) The conserved sequence block is highlighted in a red box; poly-G element following the conserved sequence block is highlighted with a red underscore. (*B*) The alignment of the 72 bp repeat among species. (*C*) The 206 bp repeat can be found prior to (copy 1) and after (copy 2) the conserved sequence block. (*D*) An ML phylogenetic reconstruction based on (*A*); nodal supports are bootstrapping values. (*E*) An ML phylogenetic tree based on (*B*). (*F*) An ML phylogeny based on (*C*). Sequences from *D. neptunus* are not included in (*A*), (*C*), (*D*), and (*F*) because they are too divergent to perform proper sequence alignment analyses with the other sequences; the sequences are available in supplementary data. gr, *D. grantii*; her, *D. hercules*; hy, *D. hyllus*; lic, *D. lichyi*; ma, *D. maya*; nep, *D. neptunus*; occ, *D. occidentalis*; pas, *D. paschoali*; ty, *D. tityus*; sep, *D. septentrionalis*.

In the control region of the ten *Dynastes* mitogenomes, our sequence alignments revealed two conserved sequence blocks (only one in *Dynastes grantii*) and one poly-G element (except in *D. neptunus*; figs. 1*C* and 2; supplementary table S1, Supplementary Material online; Saito et al. 2005; Duarte et al. 2008). The first conserved sequence block was located before the poly-G element, while the second conserved sequence block was near the 3′ end of the control region (fig. 1*C*). The lack of poly-G element associated with the second conserved sequence block in our data set is not unique, for example, a similar result has been reported in blowflies (Duarte et al. 2008). In addition, we found that before and after the conserved sequence blocks, there were A + T rich regions that can form stem-and-loop structures (fig. 2). The longest poly-G elements were observed in *D. hyllus* and *Dynastes maya* (12 bp).

There were multiple types of tandem repeats identified in the control region of the *Dynastes* mitogenomes (figs. 1 and 2). Specifically, all ten mitogenomes shared the same 72-bp tandem repeats at the 5′ end of the control region (figs. 1*C* and 2*B*; supplementary table S1, Supplementary Material online; AT contents = ca. 60%). Additionally, there were 206-bp tandem repeats (figs. 1*C* and 2*C*; the length of this repeat may vary from 203 to 207 bp; AT contents = ca. 55%) found in the middle to the 3′ end of the control region in all *Dynastes* beetles, except *D. neptunus*. For the outgroup species, *D. neptunus* (from a different subgenus, *Theogenes*, of the genus *Dynastes*), although a 206-bp tandem repeat could not be identified, there was another type of tandem repeat (the length of this repeat ranged from 195 to 197 bp) found in the control region. Importantly, closely related taxa (following the nuclear-species tree; Huang 2017) often exhibited a similar copy number of the same type of tandem repeats (fig. 1*C*; supplementary table S1, Supplementary Material online). Furthermore, we found that the 206 bp tandem repeats, including the 195-bp tandem repeats in *D. neptunus*, were separated into two sections that flank the first conserved sequence block in the control region (fig. 1*C*).

### Phylogenetic Trees

**Fig. 3.**
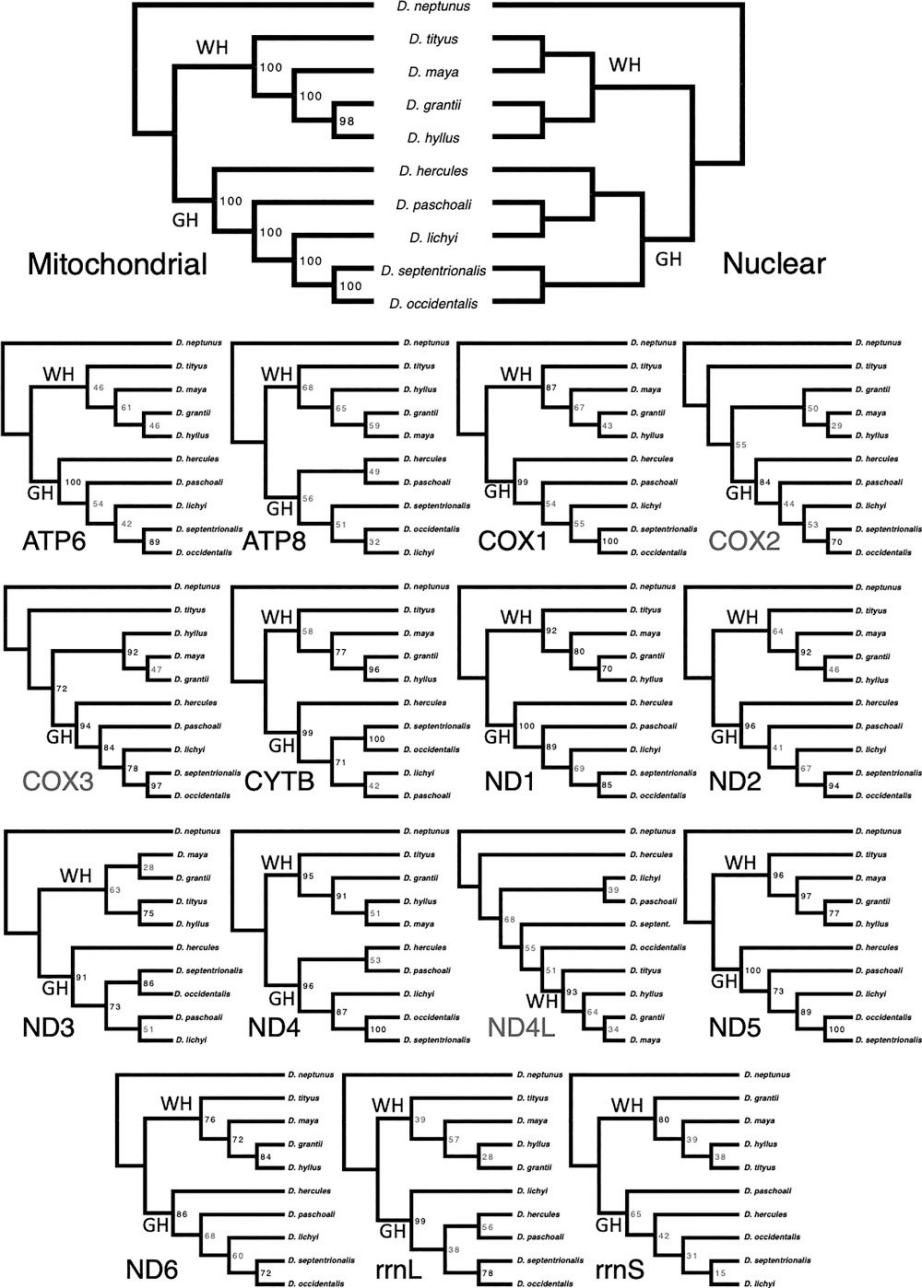
Diversification history among *Dynastes* beetles. Mitochondrial: cladogram inferred using the concatenated mitochondrial data set. Nuclear: a nuclear-genome species tree from Huang (2016a, 2017). Gene-tree topologies inferred by each individual mitochondrial locus are shown with the locus name. Numbers on the nodes are bootstrap supports. Mitochondrial gene trees that did not recover the monophyly of either Giant Hercules clade (GH) or White Hercules clade (WH) were highlighted in gray. Additionally, nodes with bootstrapping support values <70 were shown in gray.

Our phylogenetic analyses using maximum likelihood based on the concatenated mitochondrial loci data set and the best molecular evolutionary models specified for each locus (Supplementary table S2, Supplementary Material online) showed the evolutionary relationships among the *Dynastes* beetles in figure 2 (mitochondrial). All branching patterns received very high bootstrap supports. Specifically, only one node had a support value of 98; all remaining nodes were supported by a value of 100. According to a previous study, the subgenus *Dynastes* can be further subdivided into two groups: the White and the Giant Hercules groups (Huang 2017). Our concatenated mitochondrial phylogeny revealed a first split between species from the Giant Hercules (*D. hercules*, *D. lichyi*, *Dynastes paschoali*, *Dynastes occidentalis*, and *D. septentrionalis*) and species from the White Hercules (*D. maya*, *D. grantii*, *D. hyllus*, and *D. tityus*) groups, which was also supported by the topology of the nuclear-genome-based species tree (fig. 3; nuclear; the nuclear-genome-based tree was reconstructed using genome-wide SNP data under a coalescent-based model; see Huang 2017). However, the branching patterns within the Giant Hercules and those within the White Hercules groups inferred from the concatenated mitochondrial phylogeny differed from those inferences from the nuclear-genome tree (fig. 3). For example, *D. maya* and *D. tityus* were inferred to be sister species according to the nuclear-genome tree, but the newly reconstructed concatenated mitochondrial phylogeny indicated that *D. tityus* was the sister lineage to the other three White Hercules beetles.

The reconstructed individual gene trees showed various topologies with moderate-to-high bootstrap supports for the branching patterns (fig. 3). Specifically, only *ND5* inferred a gene-tree topology with all branching supports >70 (most of the nodes were supported with values >90). The *ND5* tree topology also exactly matched the inferred concatenated mitochondrial phylogeny. The *ND1* gene tree had all but one node of their branching patterns supported with >70% bootstrap values. The most commonly used *COX1* locus inferred an identical topology as the concatenated mitochondrial phylogeny, but bootstrap support values for its branching pattern were not always high (ranged from 43 to 99). All the other loci inferred gene trees, with some nodes supported by <60 bootstrap values. All the *ND* loci, except the short *ND4L* fragment, the two ribosomal RNA loci, *CYTB*, the two *ATP* loci, and the commonly used *COX1*, inferred that the Giant Hercules and White Hercules groups were reciprocal monophyletic groups. The remaining gene-tree topologies, *ATP8*, *COX2*, *COX3*, and *ND4L*, either did not recover a monophyletic White Hercules or a monophyletic Giant Hercules group.

Interestingly, we found that phylogenetic reconstructions based on the 72 bp tandem repeats and the conserved sequence block also revealed the reciprocal monophyletic relationship between Giant and White Hercules beetles (fig. 2). The 206 bp tandem repeats located before and after the conserved sequence blocks formed two phylogenetic lineages, which implied an ancient duplication event (fig. 2). Nevertheless, the phylogenetic relationships among species within Giant and within White Hercules beetles were not well resolved, where the bootstrapping support values for the branching patterns were low (fig. 2). The number of parsimony informative sites contained in these types of tandem repeats was 10 and 64 for 72 and 206 bp tandem repeats, respectively. There were nine parsimony informative sites identified in the alignment for the molecular region flanking and including the conserved sequence block (fig. 2). The amount of sequence variation, in terms of the number of parsimony informative sites, was higher for elements (e.g., the 72 bp repeats) in the control region (∼13–20% sites are parsimony informative) than the protein-coding regions of the mitogenome (∼10%; fig. 2 and supplementary fig. S12, Supplementary Material online).

### Topological and Selection Tests

Different protein-coding and rRNA loci of the *Dynastes* beetle mitogenome may or may not have inferred the same phylogenetic topology (fig. 3; table 2). Specifically, only *ATP6*, *COX1*, *ND1*, *ND2*, *ND5*, and *ND6* inferred the exact same topology as inferred by the concatenated mitochondrial phylogeny. Nevertheless, none of the mitochondrial loci statistically rejected the concatenated mitochondrial phylogeny (based on results from Shimodaira–Hasegawa [SH] and approximately unbiased [AU] tests; table 2). On the other hand, none of the mitochondrial loci inferred the exact same topology as the nuclear-genome tree (fig. 3; table 2); furthermore, many mitochondrial protein-coding loci statistically rejected the topology of the nuclear-genome tree based on the SH and AU test results (table 2). The loci that statistically rejected the topology of the nuclear-genome tree were *ATP8* (AU only), *COX2* (AU only), *COX3* (AU only), *ND1* (SH and AU), *ND2* (SH and AU), *ND4* (SH and AU), and *ND5* (SH and AU). Note that the length of the sequence alignments was correlated with the number of parsimoniously informative sites (fig. S12); however, the number of parsimoniously informative sites did not relate to the statistical support for rejecting the nuclear-species tree topology (fig. S13). Interestingly, the most frequently used locus for phylogenetics, phylogeography, and species delimitation—*COX1—*inferred the same topology as the concatenated mitochondrial phylogeny (fig. 3) but did not statistically reject the nuclear-genome tree. We also reconstructed a maximum likelihood phylogeny based on the *COX1* barcoding region using the newly sequenced data and data from a previous study (Huang and Knowles 2016). The newly sequenced individuals clustered with individuals of their corresponding species complexes (Huang 2017) with high bootstrap support values, indicating that our identification of the species and the sequencing quality of the new results were robust (supplementary fig. S14, Supplementary Material online).

**Table 2:** Summary Statistics of Protein-coding and Ribosomal RNA Genes for Phylogenetic Utility

Gene	Length	d*n*/d*s*	P_site_^a^	rf_nuc_^b^	rf_mt_c	SH_nuc_^d^	SH_mt_^e^	AU_nuc_^f^	AU_mt_^g^
ATP6	672	0.04036	82	6	0	0.147	1	0.123	0.877
ATP8	156	0.09067	13	10	6	0.0873	1	**0**.**0455**	0.954
COX1	1 536	0.00423	162	6	0	0.173	1	0.156	0.844
COX2	684	0.02454	63	10	4	0.0581	1	**0**.**0377**	0.962
COX3	786	0.01971	89	10	4	**0**.**025**	1	**0**.**0151**	0.985
CYTB	1 143	0.02283	133	4	2	0.314	1	0.348	0.652
ND1	945	0.01285	111	6	0	**0**.**042**	1	**0**.**0385**	0.962
ND2	1 011	0.05026	109	6	0	**0**.**0195**	1	**0**.**00724**	0.993
ND3	345	0.06997	32	6	6	0.433	1	0.439	0.561
ND4	1 338	0.02944	157	8	4	**0**.**0216**	1	**0**.**0159**	0.984
ND4L	291	0.00370	27	10	10	0.302	1	0.313	0.687
ND5	1 713	0.02278	189	6	0	**0**.**0133**	1	**0**.**00528**	0.995
ND6	474	0.04902	74	6	0	0.0865	1	0.0583	0.942
rrnL	1 227	NA	59	6	4	0.0874	1	0.0623	0.938
rrnS	677	NA	21	10	8	0.201	1	0.179	0.812

Note.—^a^Number of parsimoniously informative sites.

b Robinson–Foulds distance calculated using the species-tree topology (Huang 2016a).

c Robinson–Foulds distance calculated using the concatenated mitochondrial tree.

d Results from Shimodaira–Hasegawa test using the species-tree topology.

e Results from Shimodaira–Hasegawa test using the concatenated mitochondrial tree.

f Results from approximately unbiased test using the species-tree topology.

g Results from an approximately unbiased test using the concatenated mitochondrial tree.

*P* values < 0.05 were highlighted in bold.

All 13 protein-coding genes had an estimated d*n*/d*s* ratio smaller than 0.1 (table 1), indicating purifying selection. *ATP8* had the highest estimated d*n*/d*s* ratio (0.0906), whereas *ND4L* had the lowest estimated ratio (0.0037), followed by *COX1* (0.0042). The estimated value of the d*n*/d*s* ratio did not correlate with the length of the alignment nor the number of parsimoniously informative sites. Similarly, the estimated value of the d*n*/d*s* ratio did not correlate with the calculated Robinson–Foulds value nor the statistical significance of the topology tests for either the mitochondrial concatenated tree or the topologies of the nuclear-genome species tree (supplementary fig. S15, Supplementary Material online).

## Discussion

Our results demonstrated that the gene arrangement of the mitogenome has remained constant in *Dynastes* beetles, where the arrangement is the same as the reconstructed ancestral insect mitogenome (Cameron 2014; fig. 1*A*). On the other hand, the control region evolved rapidly and the size of the mitogenome varied among lineages (figs. 1*C* and 2; supplementary table S1, Supplementary Material online). Specifically, by using long-read sequencing data, we were able to confidently assemble complete control regions of the ten mitogenomes, which would not have been possible if only short-read sequencing data were applied (supplementary figs. S1—S11, Supplementary material online). We further showed that variation in the copy numbers of two types of tandem repeats may have contributed to the different mitogenome sizes among *Dynastes* beetles (fig. 1*C*; supplementary table S1, Supplementary Material online), where the newly sequenced circular mitogenomes were among the largest ones in animals, particularly in insects. Importantly, the flanking regions of the conserved sequence blocks in the control region were found to be dominated by a specific type of tandem repeats implying functional evolution.

Our phylogenetic analyses further showed that, although the gene-tree topologies inferred using different mitochondrial loci were not identical (fig. 3; table 2), the phylogenetic information contained in the loci, both protein-coding and ribosomal RNA, did not statistically reject the concatenated mitochondrial phylogeny. We, on the other hand, obtained statistically significant results of mito-nuclear phylogenetic discordance from about 50% of the mitochondrial protein-coding loci. The results of mito-nuclear discordance were most apparent when using loci from the *NADH dehydrogenase* gene family. In the following sections, we discuss the application of long-read sequencing technology to sequencing and assembling mitogenomes, the evolution of the mitogenome with a particular emphasis on the control region in Hercules beetles, the phylogenetic discordances among genetic data sets, and the phylogenetic utility of mitochondrial DNA in animal molecular phylogenetics.

### Long-read Sequencing Revealed Rapidly Evolving Mitogenome Size via Copy-number Variation in Tandem Repeats in Dynastes Beetles

In the *Dynastes* beetle system, we unveiled mitogenomes of a size that among animals, and particularly insects, was uncommonly reported (Boyce et al. 1989; Lavrov and Pett 2016; Desalle et al. 2017; Filipović et al. 2021). Specifically, while the size of a circular animal mitogenome can be >40 kb, the size of most circular animal mitogenomes are <20 kb (Desalles et al. 2017). While a tube worm has the largest linear mitogenome reported to date (>80 kb; Stampar et al. 2019), a recent study has reported a >90 kb circular mitogenome in a cnidarian species (Novosolov et al. 2022). In insects, the mitogenome size has often been reported to be around 15–16 kb (Cameron 2014; Desalle et al. 2017; but exceptionally large mitogenomes have been found in bark weevils; Boyce et al. 1989). However, when long-read sequencing approaches were applied in recent years, the reported mitogenome sizes were often >20 kb (e.g., Sayadi et al. 2017; Filipović et al. 2021). Nevertheless, our reported *Dynastes* mitogenomes remain among the largest sized beetle mitogenomes published to date.

We infer that this large size is a result of a phenomenon uncommonly investigated in insect mitochondria: rapid tandem-repeat evolution (Yuan et al. 2015; Sayadi et al. 2017; Filipović et al. 2021). Specifically, in the *Dynastes* beetle system, a recent study that employed only the short-read sequencing method (Illumina MiSeq) reported a complete mitogenome assembly of *D. hercules* with a total length of 17 813 bp, where the control region was estimated to be 3 031 bp long (Ayivi et al. 2021). However, with estimations of 60 replicates of a 72-bp repeat (ca., 4 320 bp in total length) and 20 replicates of a 206-bp repeat (ca., 4 120 bp in total length), our assembly, with the help from long-read sequencing data for the same species, *D. hercules*, resulted in a mitogenome size of 25 542 bp (fig. 1*B*), which is approximately 8 000 bp longer than the one that did not specifically account for repeats using long-read sequencing technology (cf., Ayivi et al. 2021). Similar estimates of 15–16 kb mitogenome sizes could result when only short-read sequencing data were used for assemblies in other *Dynastes* species using our data sets (Supplementary fig. S11, Supplementary Material online). Our results thus support a recent argument that the growing accumulation of published mitogenomes that only utilized short-read sequencing data likely failed to properly assemble highly repetitive regions, such as the control region, and the genomic assemblies were likely incomplete, even if some of them had been claimed to be complete (Filipović et al. 2021; Formenti et al. 2021; Kinkar et al. 2021).

The ramification of neglecting an important evolutionary feature in the mitogenome—the evolution of tandem repeats in the control region, which makes up about 30% of the genome size in *Dynastes* beetles—may seem minimal at first for animal phylogenetics and systematics, because most studies have used protein-coding genes, and/or the ribosomal rRNA loci, to infer phylogenetic trees (e.g., Łukasik et al. 2019). However, variation in repeat number of tandem repeats has been demonstrated to have evolutionary implications (Melters et al. 2013; Omote et al. 2013; Yuan et al. 2015; Bronstein et al. 2018), and new models and computer programs have been developed to leverage such data and information for evolutionary inferences (e.g., Schull et al. 2022). Our data also showed that the sequence of the tandem repeats can be used to successfully recapitulate the evolutionary history among *Dynastes* beetles (fig. 2). Moreover, a duplication event of the 206 bp tandem repeats may have occurred before the diversification of *Dynastes* beetles, and a secondary duplication following a loss of one of the original 206 bp tandem repeats may have occurred in the species *D. lichyi*. Note that we could not identify homologs of the two newly identified tandem repeats in other insects, even though tandem repeats have been reported in the control region from many other insects (Yuan et al. 2015; Sayadi et al. 2017). Our results thus imply that the phylogenetic utility of tandem repeats in the control region may be limited to recent divergences. On the other hand, the variation of copy number and a higher DNA sequence evolution rate of the tandem repeats can be of potential application for future population genetic studies.

Furthermore, tandem repeats have been an interesting evolutionary phenomenon in the control region of animal mitochondria not only because of their phylogenetic and systematic utilities (Faber and Stepien 1997; Mundy and Helbig 2004; Wan et al. 2012; Wang et al. 2015; Novosolov et al. 2022), but also because of their evolutionary dynamics and the implication on mitochondrial function (Mundy and Helbig 2004; Saito et al. 2005; Yuan et al. 2015; Chen et al. 2020; Kinkar et al. 2021; Novosolov et al. 2022). For example, we found that in *Dynastes* mitogenomes, the 206 bp tandem repeats were closely located both prior to and after the conserved sequence blocks. The conserved sequence blocks are believed to be involved in mtDNA transcription and replication processes (Zhang and Hewitt 1997; Saito et al. 2005). As a result, the rapid evolution in the control region of *Dynastes* mitogenomes via copy-number variation in tandem repeats may have impacted mitochondrial function. As long-read sequencing technology advances, the mitogenome sequencing and assembling field, or industry, can and should move on to provide improved complete mitogenomes. Future studies that focus not only on systematics, but beyond systematics, can then design their research by relying on unbiased mitogenome assemblies.

### Mitochondrial Genealogy, Gene Trees, and Mito-nuclear Phylogenetic Discordance

We found that none of the mitochondrial protein-coding or rRNA loci statistically rejected the concatenated mitochondrial phylogeny, supporting the hypothesis that they share a common genealogical history. Consequently, the common practice of concatenating mitochondrial-sequence data to infer phylogenetic relationships among taxa seems justified (Boore 1999; Rubinoff and Holland 2005; Wan et al. 2012; Cameron 2014; Ladoukakis and Zouros 2017; Łukasik et al. 2019; Tang et al. 2019; Zhang et al. 2021). Our results also showed that about half of the mitochondrial protein-coding loci (6 out of 13) inferred an identical topology as the concatenated mitochondrial tree; furthermore, 10 of the 13 protein-coding loci and the two rRNA loci supported the reciprocal monophyly between the White and Giant Hercules groups. Those that inferred the identical tree topology, however, did not have statistically significant longer sequence data (*t* = 1.469; *P* = 0.1715). Similarly, those loci that did and did not support reciprocal monophyly between the White and Giant Hercules groups had similar sequence lengths (*t* = 1.5549; *P* = 0.1624). Because the number of informative sites for phylogenetic study was correlated with the length of the sequence alignment in our data set (supplementary fig. S12, Supplementary Material online), we argue that the differences in topological inference were not due to the quantity of phylogenetic information contained in different loci. In addition to the effect of sequence length, we also demonstrated that this variation in phylogenetic-tree topology was not associated with the strength of purifying section (table 2). That is, even though there is statistical agreement that there is a common mitochondrial genealogy among mitochondrial loci, there are still locus-specific evolutionary nuances that could not be attributed to the length of the locus nor the strength of selection.

Idiosyncrasies in mitochondrial gene-tree topology, and in the branch supports for different topologies, can have impacts on molecular-based studies of biodiversity. For example, one commonly asked question regards which locus, or loci, to use for phylogenetic reconstruction in the face of limited funding (Hebert et al. 2003; Keller et al. 2015). The question applies to taxonomic investigations (Rubinoff and Holland 2005; Pons et al. 2006), metabarcoding and barcoding studies (Hebert et al. 2003; Luo et al. 2011), and community phylogenetics (Rubinoff and Holland 2005; Boyle and Adamowicz 2015; Keller et al. 2015). We showed that the *COX1* gene tree may not be the best candidate representing mitochondrial evolutionary history, unlike in some previous studies (e.g., Luo et al. 2011). Specifically, there are five other mitochondrial loci that inferred the same phylogenetic topology, while the branching pattern of the topology was better supported using the *ND* loci (especially, *ND5*; fig. 3). Furthermore, when it comes to rejecting an alternative phylogenetic hypothesis, the phylogenetic information contained in *COX1* did not have statistical power (table 2), even though *COX1* was among the longest mitochondrial loci that had a relatively higher number of parsimoniously informative sites (table 2; supplementary fig. S12, Supplementary Material online). Note that we are not arguing that the concatenated mitochondrial-tree topology is the true evolutionary history among *Dynastes* beetles. Similarly, we are not arguing that *COX1* is a bad locus for systematics and phylogenetics. Instead, we are emphasizing that different linked loci, even if they share the same genealogical history, may still infer different phylogenetic trees with different levels of statistical power to either support or reject certain phylogenetic hypotheses. The cause for such a locus-specific idiosyncrasy could not be properly identified in this study, but the impact on phylogenetic inference is apparent. When resources are limited, we argue that *ND5*, which was a previously commonly used mitochondrial locus for ground beetle phylogeography and phylogenetic study (e.g., Sota and Nagata 2008), is the best candidate for the study of molecular biodiversity using mitochondrial loci in terms of branching supports and the statistical power associated with the data to reject alternative phylogenetic hypotheses. There are also other loci, such as *ND1* and *ND2*, that are better than *COX1* in terms of rejecting alternative phylogenetic hypotheses. Finally, *ATP6* and *ND6* appear to be at least as good as *COX1* and can infer the same tree topology. Similar arguments have also been made in vertebrates, and especially in birds (Zardoya and Meyer 1996; Paton and Baker 2006).

Our phylogenetic results demonstrated different evolutionary histories between those inferred using mtDNA and the nuclear-genome-based species tree (fig. 3). Previous studies have documented widespread mito-nuclear phylogenetic discordance throughout animal lineages (Shaw 2002; Funk and Omland 2003; Rubinoff and Holland 2005; Spinks and Shaffer 2009; Fisher-Reid and Wiens 2011; Toews and Brelsford 2012; Yan et al. 2019; Piccinini et al. 2021), while our study further demonstrated that none of the mitochondrial protein-coding and rRNA loci inferred the same topology as the nuclear-genome tree, implying that such a pattern is widespread across mitochondrial loci. However, only some mitochondrial protein-coding loci statistically rejected the nuclear-genome tree (table 2), which is partially in agreement with a previous study stating that the discordance identified may not always be statistically significant (Fisher-Reid and Wiens 2011). As mentioned in the previous section, the statistical power to reject the nuclear-genome tree did not correlate with the length of the gene, the number of parsimoniously informative sites, and the strength of purifying selection (table 2; supplementary figs. S13 and S15, Supplementary Material online). Furthermore, we found that not all loci that inferred an identical topology to the concatenated mitochondrial tree rejected the nuclear-genome tree (e.g., *COX1*) and that some mitochondrial loci that inferred a different topology to the concatenated mitochondrial tree statistically rejected the nuclear-genome phylogeny (*COX3* and *ND4*). As a result, we can only say that mito-nuclear phylogenetic discordance is a dominant pattern across mitochondrial loci in *Dynastes* beetles, but the statistical power to detect such a discordance may vary among loci.

The ecological and evolutionary causes generating mito-nuclear phylogenetic discordances may be the different rates of lineage sorting due to differences in effective population sizes between mitochondrial and nuclear genomes and to introgression between geographically proximate species in animals (Shaw 2002; Spinks and Shaffer 2009). Both historical processes could have impacted the mito-nuclear phylogenetic discordance observed in our Hercules beetle system. For example, it has been shown that introgression occurred between parapatric Hercules beetles, irrespective of the degree of phylogenetic divergence or the phenotypic similarity (Huang 2016a; Huang 2020). In this specific case, where all the studied *Dynastes* beetles were allopatrically distributed (see Huang and Knowles 2016 for a geographical distributional map), a more likely historical cause for the observed discordance would be rapid diversification. The divergences in *Dynastes* beetles occurred recently, where 15 species were generated within the past 3 million years as a result of habitat fragmentation and new ecological opportunity created under new climatic conditions (Huang 2016b). Because the nuclear-genome tree accounted for the stochastic coalescent process across randomly sampled loci from the genome using multiple individuals per species (Huang 2016a, 2017), we believe that the nuclear-genome tree better represents the diversification history of the *Dynastes* beetles. The nuclear-genome tree also makes better sense in terms of morphological similarity and geographic distribution among *Dynastes* beetles (Huang 2017). Nevertheless, mtDNA and the phylogenetic inferences made from mtDNA, from a single locus or the concatenated data set, still provides a unique snapshot of what a genealogy could be like after rapid diversification with a much smaller effective population size.

## Conclusion

The structure and size of the mitochondrial control region is highly variable in *Dynastes* beetles because of the copy-number evolution in two types of tandem repeats. However, tandem-repeat evolution could have gone undetected if only short-read sequencing approaches were used. We argue that to announce a “complete” mitogenome assembly, one should include long-read sequencing data given the difficulty in assembling highly repetitive regions using only short-read data sets. There is a rapid accumulation of mitogenomes, either deemed as complete or almost complete, because of the application of next-generation sequencing. However, because of the reliance on short-read sequencing data, an important mitogenome feature, tandem repeats, that may have played an important role in systematics and molecular evolution may become neglected. Specifically, the copy-number and sequence variations of the tandem repeats, although may not be suitable for species-level phylogenetic reconstruction, may be good candidates for future population genetic studies. We also demonstrated that, compared with *COX1*, multiple *ND* loci could be better mitochondrial candidates for molecular-phylogenetic studies. Nevertheless, because of the scope of this study, such a drastic impact of long-read sequencing data on the resulting size of mitogenome assemblies, the high variability of the mitochondrial control region, and the idiosyncratic phylogenetic inference among mitochondrial loci may only apply to Hercules beetles. Additional investigations are undoubtedly needed to objectively assess the effects on mitogenome assemblies when applying different sequencing technologies and the phylogenetic utility of different loci.

## Materials and Methods

### Sequencing and Assembly

Adult male beetles of *D. grantii*, *D. hyllus*, and *D. tityus* were collected in the years of 2018, 2019, and 2018 from Payson, AZ, USA; Oaxaca, Mexico; and NC, USA, respectively. Adult males of *D. hercules*, *D. maya*, and *D. neptunus* and larvae of *D. lichyi*, *D. occidentalis*, *D. septentrionalis*, and *D. paschoali* were obtained using captive-bred pet beetle samples. These specimens were derived from progenies originally from Guadeloupe island; Chiapas, Mexico; Ecuador, Ecuador, Ecuador; Nicaragua; and Bahia, Brazil, respectively. Detailed geographic origins for the captive-bred samples were unfortunately unavailable. Taxonomically, *D. grantii*, *D. hyllus*, *D. tityus*, and *D. maya* belong to the White Hercules group, whereas *D. hercules*, *D. lichyi*, *D. paschoali*, *D. occidentalis*, and *D. septentrionalis* belong to the Giant Hercules group (Huang 2017). *Dynastes neptunus* belongs to a different subgenus, *Theogenes*, of the genus *Dynastes* (Huang 2017). Genomic DNA was extracted from representatives of ten *Dynastes* species using the DNeasy Blood and Tissue Kit (QIAGEN). Library preparation was completed using the KAPA Hyper Prep Kit KK8502 and the Oxford Nanopore Technologies (ONT) Ligation Sequencing Kit (SQK-LSK109). Long-read sequencing was conducted on an ONT MinION Mk1B unit using FLO-MIN 106D, R9 version flow cells. Additionally, short-read sequencing was applied to the DNA extracted from the same individuals using a NovaSeq 6000 machine (150 bp pair-end sequencing) by the GENOMICS company (Taipei, Taiwan). The total amount of molecular data generated by both sequencing technologies for each sample is summarized in Table 1.

Raw nanopore reads were base-called using Guppy (v4.0.11) and filtered to include only reads above 1 kb in length and with an average *q* score above 10 using NanoFilt (v2.6.0, De Coster 2018). Mitogenome assembly was conducted in two rounds. For the first round, non-mitochondrial reads were removed using mtBlaster (Franco-Sierra and Díaz-Nieto 2020). MtBlaster uses a reference sequence to compare with candidate reads, and in round 1 a *Popillia japonica* mitochondrion (NCBI: NC_038115.1) reference was used. This reduced set of reads was then assembled using minimap2 (v2.17, Li 2018) and miniasm (v0.3, Li 2016). If the resulting draft assembly contained more than one contig, a blast search was conducted to confirm which contig contained the COX1 gene, and only that contig was retained. Minipolish (v0.1.3, Wick and Holt 2019) was then used to cleanup assembly errors using the reads output by mtBlaster, concluding assembly round 1. Assembly round 2 was identical except that in the mtBlaster step, the reference sequence used was the polished assembly result from round 1. Following the second round, Illumina short reads were aligned to the assembly using Bowtie 2 (v2.4.1, Langmead and Salzberg 2012). The resulting alignment was then used to polish the assembly again using Pilon (v1.23, Walker 2014). Each final circular assembly was rotated to begin at the COX1 gene. We further used the Tandem Repeats Finder (v4.09; Benson 1999) to identify all types of tandem repeats in the assembled mitogenomes with default settings.

Furthermore, the control region is known as the most rapidly evolving part of the mitogenome in most insects (Dotson and Beard 2001; Cameron 2014). In this study, we used Geneious Prime 2020.1.2 (https://www.geneious.com/) to align and inspect the control regions of the ten newly assembled *Dynastes* mitogenomes. Specifically, we looked for conserved sequenced blocks and poly-G elements in the mitogenomes because their position in relation to stem-and-loop structure and copy-number variation may impact mtDNA transcription and replication processes in insects (Zhang and Hewitt 1997; Saito et al. 2005).

### Tree Building and Topology Tests

MitoFinder (v1.4, Allio et al. 2020) was used to annotate the assembled and polished mitogenomes using an annotated *O. rhinoceros* mitogenome (GenBank: MT457815.1) as a reference. Kalign (v3.3, Lassmann and Sonnhammer 2005) was used to align corresponding genes from each of the ten Hercules beetle species, and ModelTest-NG (v0.1.6, Darriba et al. 2020) was used to select optimal nucleotide-substitution models. Alignments generated for the individual locus were subsequently concatenated using SequenceMatrix (v1.8.2, Vaidya et al. 2011). The concatenated data set was then input to RAxML-NG (v1.0.1; Kozlov et al. 2019) to estimate the phylogeny, with all substitution models for different gene partitions set to the best-fitting molecular-evolution model. *Dynastes neptunus* (subgenus *Theogenes*) was assigned as an outgroup to polarize the tree topologies. Furthermore, we inferred individual gene trees for each locus, including the two types of tandem repeats (see Results section), via PhyML 3.0 (Guindon et al. 2010) with smart model selection (SMS option; Lefort et al. 2017). The supports for branching patterns in each gene-tree topology were evaluated using 100 bootstrap replicates.

IQ-TREE 2 (v2.1.2, Minh et al. 2020) was used to conduct the SH (Shimodaira and Hasegawa 1999) test and the AU(Shimodaira 2002) test to quantify discordance between tree topologies. A third measure of topological distance, the Robinson–Foulds metric (RF; Robinson and Foulds 1981), was calculated using the RF.dist() function in the phangorn R package (Schliep 2011). Each locus was measured with these three tests against both a nuclear-genome-tree topology (retrieved from Huang 2017) and the concatenated mitochondrial phylogeny generated in this study.

### Test for Positive/Purifying Selection

Selection pressure (d*n*/d*s*) for each gene was calculated using PAML (v4.8, Yang 2007), given the individual gene alignments against both the topology of the nuclear-species tree (Huang 2017) and the topology of the concatenated mitogenome. A d*n*/d*s* ratio >1 indicates positive selection, while those <1 indicate purifying selection.

## Supplementary Material

evac147_Supplementary_Data

## Data Availability

The assembled and annotated mitogenomes have been archived in GenBank with the following accession numbers: ON312096–ON312105. The raw reads from nanopore and Illumina sequencing results were archived under BioProject: PRJNA815811. Alignment files for protein-coding and ribosomal RNA loci were provided as supplementary files.
