# Supplementary material for: Long-read Sequencing Data Reveals Dynamic Evolution of Mitochondrial Genome Size and the Phylogenetic Utility of Mitochondrial DNA in Hercules Beetles (Dynastes; Scarabaeidae)
Source: Genome Biol Evol. 2022 Sep 29;14(10):evac147. doi: 10.1093/gbe/evac147 (PMC9576211; doi:10.1093/gbe/evac147)
Supplement: evac147_Supplementary_Data [file evac147_supplementary_data.docx]

Table S1. The position of the tandem repeats, poly-G elements, and conserved sequence blocks (CBS) in the control region of *Dynastes* mitogenomes.

| Species | 72 bp (No. of repeats) | 206 bp (No. of repeats) | CBS | 206 bp (No. of repeats) | Poly-G elements |
| --- | --- | --- | --- | --- | --- |
| *D. tityus* | 209-5527 (74) | 5531-6916 (7) | 8176-8191 13196-13210 | 8678-11934 (16) | 8204-8213 |
| *D. maya* | 221-4509 (60) | 4516-6306 (9) | 7553-7568  12264-12278 | 7718-11008 (16) | 7582-7596 |
| *D. hyllus* | 206-3034 (39) | 3038-4414 (7) | 5661-5676  9362-9376 | 6012-8194 (10) | 5691-5704 |
| *D. grantii* | 206-3317 (43) | 3320-4907 (8) | 6156-6171 | 6544-8976 (12) | 6186-6197 |
| *D. septentrionalis* | 200-2999 (38) | 2995-4572 (8) | 5831-5846  9956-9970 | 6245-8694 (12) | 5860-5872 |
| *D. occidentalis* | 199-2993 (39) | 2989-4566 (8) | 5825-5839  9723-9737 | 6239-8463 (11) | 5854-5865 |
| *D. hercules* | 201-4606 (60) | 4605-5984 (7) | 7231-7246  10715-10729 | 7658-9459 (9) | 7261-7271 |
| *D. lichyi* | 195-3410 (45) | 3413-4784 (7) | 6043-6058  9532-9546 | 6513-8272 (9) | 6072-6084 |
| *D. paschoali* | 211-4237 (56) | 4233-5810 (8) | 7069-7084  10770-10784 | 7296-9506 (11) | 7098-7109 |
| *D. neptunus* | 188-1817 (23) | 1981-3957* (7) | 5209-5220  8868-8879 | 5650-7617* (10) | NA |

*The length of the 206 bp tandem repeats in *Dynastes neptunus* is actually 195 bp, which differs from those of the other *Dynastes* beetles.

Table S2. The best-fitting molecular-evolution models for the mitochondrial loci.

| Locus | Best-fit model | lnL |
| --- | --- | --- |
| *ATP6* | GTR+FO+G4m | -1930.609042 |
| *ATP8* | HKY+FO+G4m | -402.539627 |
| *COX1* | GTR+FO+I+G4m | -4296.610220 |
| *COX2* | HKY+FO+I+G4m | -1817.457820 |
| *COX3* | GTR+FO+I | -2226.543981 |
| *CYTB* | HKY+FO+I+G4m | -3290.290207 |
| *ND1* | HKY+FO+I+G4m | -2614.156299 |
| *ND2* | HKY+FO+G4m | -2797.036299 |
| *ND3* | GTR+FO+I | -902.995975 |
| *ND4* | HKY+FO+I+G4m | -3728.690072 |
| *ND4L* | HKY+FO+G4m | -662.514829 |
| *ND5* | GTR+FO+I+G4m | -4755.338465 |
| *ND6* | GTR+FO+I | -1462.386485 |
| rrnL | GTR+FO+I+G4m | -2533.293116 |
| rrnS | HKY+FO+I | -1255.984862 |

Table S3. Collection and voucher information for the studied samples.

| Species | Collection information | Voucher information* |
| --- | --- | --- |
| *Dynastes tityus* | Fayetteville, North Carolina, USA; June/2018; adult male | Specimen: BL_0003; DNA: Dty. |
| *Dynastes maya* | Captive bred adult male from a progeny of a wild female from Chiapas, Mexico; May/2019 | Specimen: BL_0094; DNA: DmaRef. |
| *Dynastes grantii* | Payson, Arizona, USA; August/2018; adult male | Specimen: BL_0001; DNA: DgAZ. |
| *Dynastes hyllus* | Santa Cruz Xoxocotlán, Oaxaca, Mexico; November/2019; adult male | Specimen: BL_0122; DNA: DhyOA6. |
| *Dynastes occidentalis* | Captive bred first instar larva from a captive bred progeny originally from Ecuador; July/2019 | Specimen: BL_002; DNA: Docc. |
| *Dynastes septentrionalis* | Captive bred first instar larva from a captive bred progeny originally from Nicaragua; July/2019 | Specimen: BL_0042; Dsep. |
| *Dynastes hercules* | Captive bred adult male from a captive bred progeny originally from Guadeloupe island. | Specimen: BL_0028; DNA: Dhh_cb. |
| *Dynastes lichyi* | Captive bred second instar larva from a captive bred progeny originally from Ecuador; December/2019 | Specimen: BL_236; DNA: Dlic. |
| *Dynastes paschoali* | Captive bred third instar larva from a captive bred progeny originally from Bahia, Brazil; July/2019 | Specimen: BL_0481; DNA: Dpas. |
| *Dynastes neptunus* | Captive bred adult male from a captive bred progeny originally from Ecuador; December/2019 | Specimen: BL_336; DNA: Dnep. |

*The collection codes for the physical specimens and DNA extracts used in the BEACHEN lab. The physical specimens and DNA extracts were stored in a -80ºC and a -20ºC freezers, respectively in the BEACHEN lab at the Biodiversity Research Center, Academia Sinica.


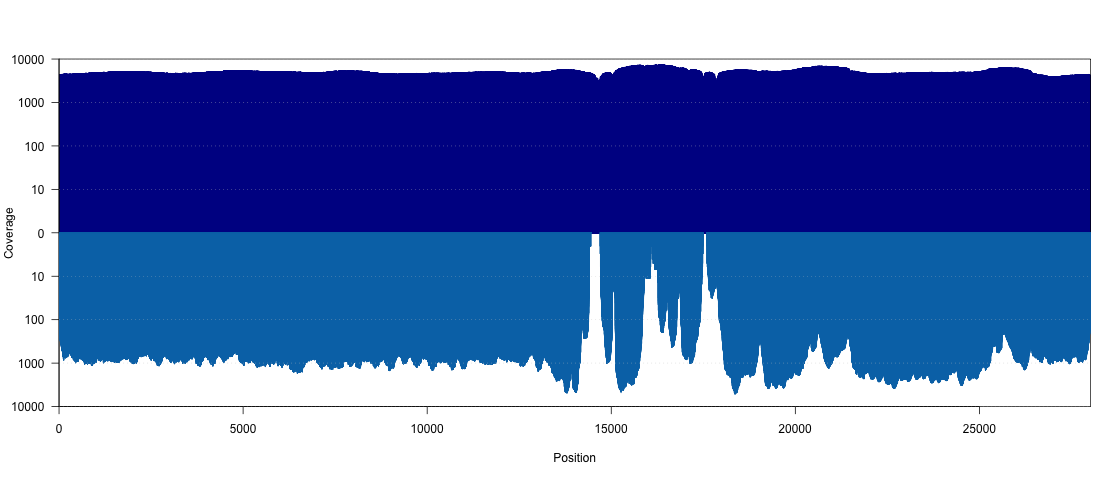


Figure S1. The estimated coverage depths from nanopore (upper dark blue) and Illumina (lower light blue) sequencing reads for *Dynastes* *tityus* mitogenome.


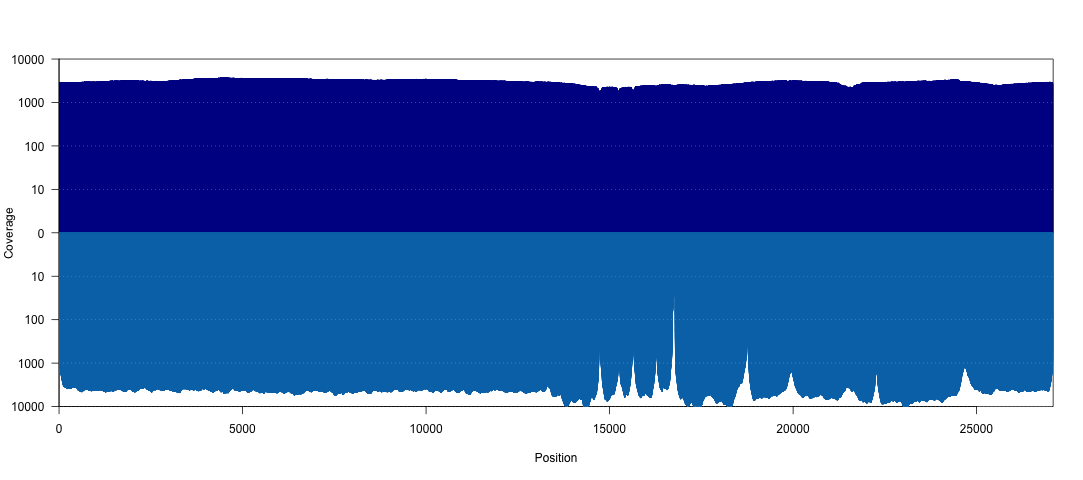


Figure S2. The estimated coverage depths from nanopore (upper dark blue) and Illumina (lower light blue) sequencing reads for *Dynastes* *maya* mitogenome.


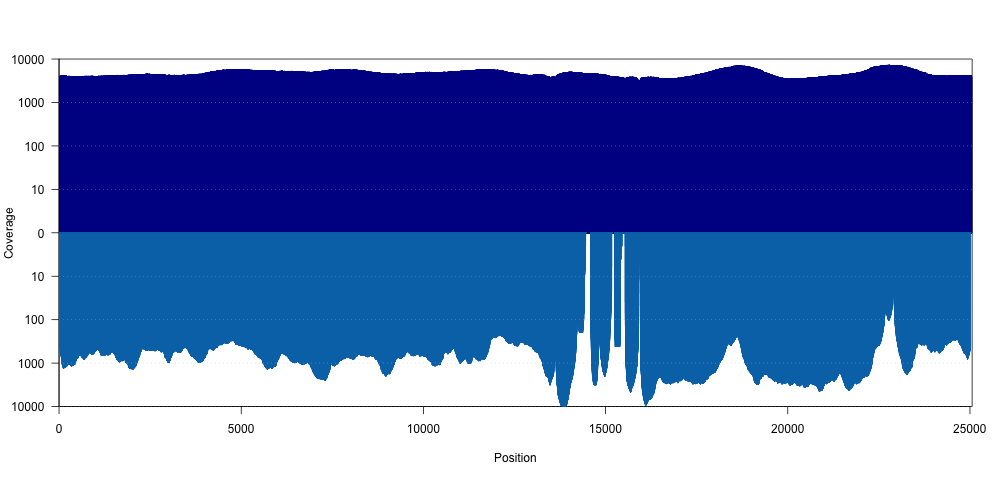


Figure S3. The estimated coverage depths from nanopore (upper dark blue) and Illumina (lower light blue) sequencing reads for *Dynastes* *grantii* mitogenome.


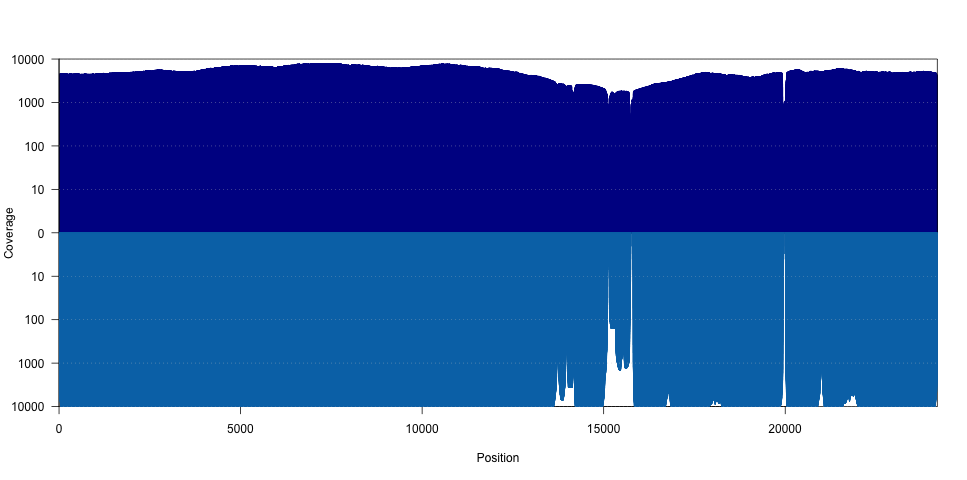


Figure S4. The estimated coverage depths from nanopore (upper dark blue) and Illumina (lower light blue) sequencing reads for *Dynastes* *hyllus* mitogenome.


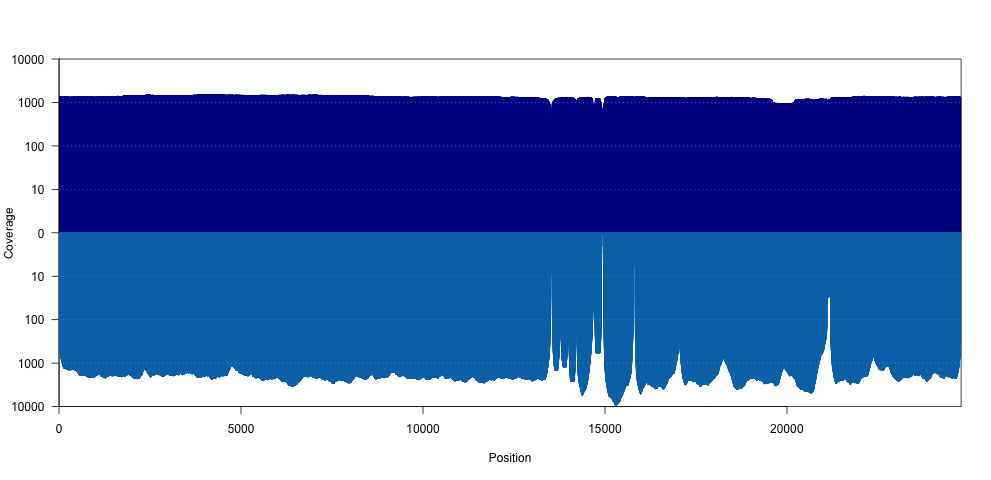


Figure S5. The estimated coverage depths from nanopore (upper dark blue) and Illumina (lower light blue) sequencing reads for *Dynastes* *septentionalis* mitogenome.


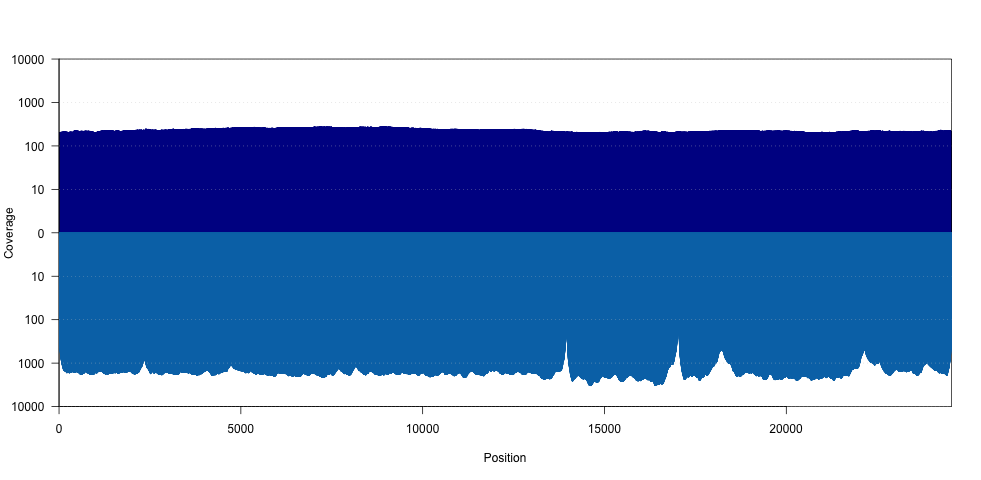


Figure S6. The estimated coverage depths from nanopore (upper dark blue) and Illumina (lower light blue) sequencing reads for *Dynastes* *occidentlais* mitogenome.


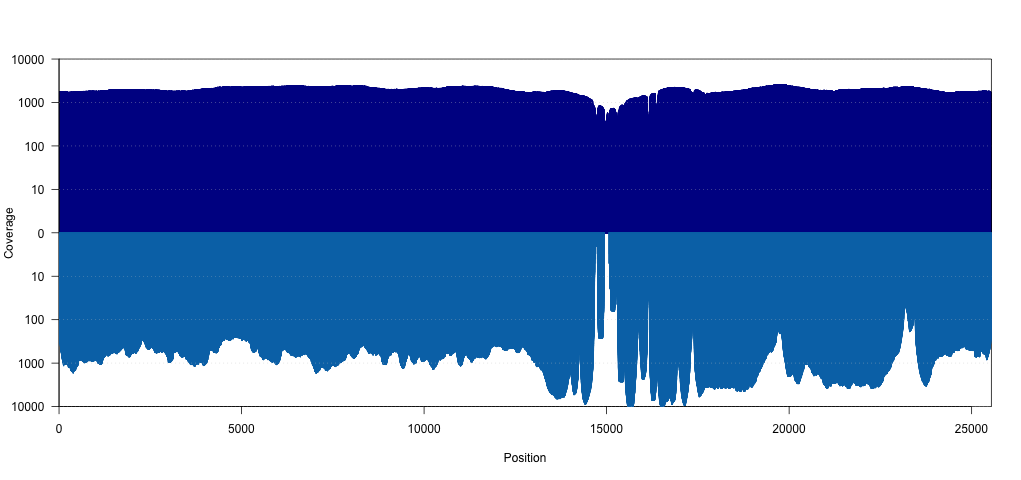


Figure S7. The estimated coverage depths from nanopore (upper dark blue) and Illumina (lower light blue) sequencing reads for *Dynastes* *hercules* mitogenome.


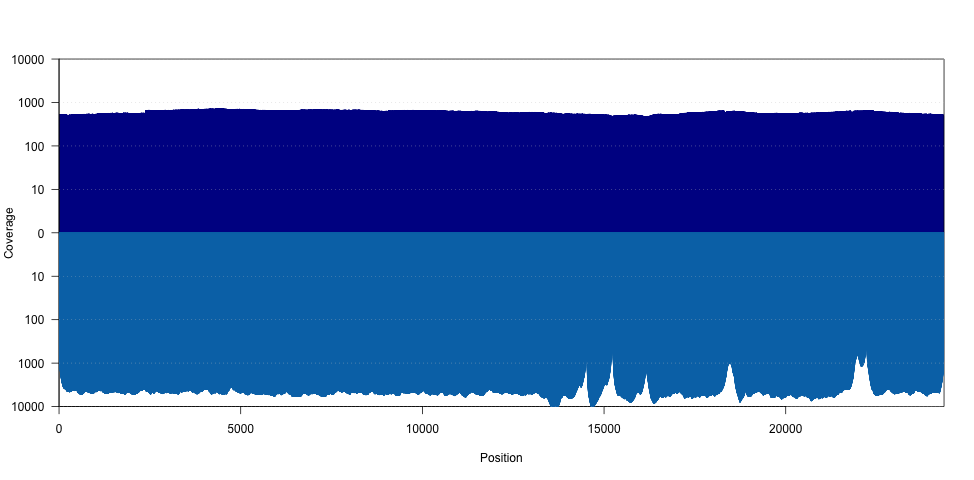


Figure S8. The estimated coverage depths from nanopore (upper dark blue) and Illumina (lower light blue) sequencing reads for *Dynastes* *lichyi* mitogenome.


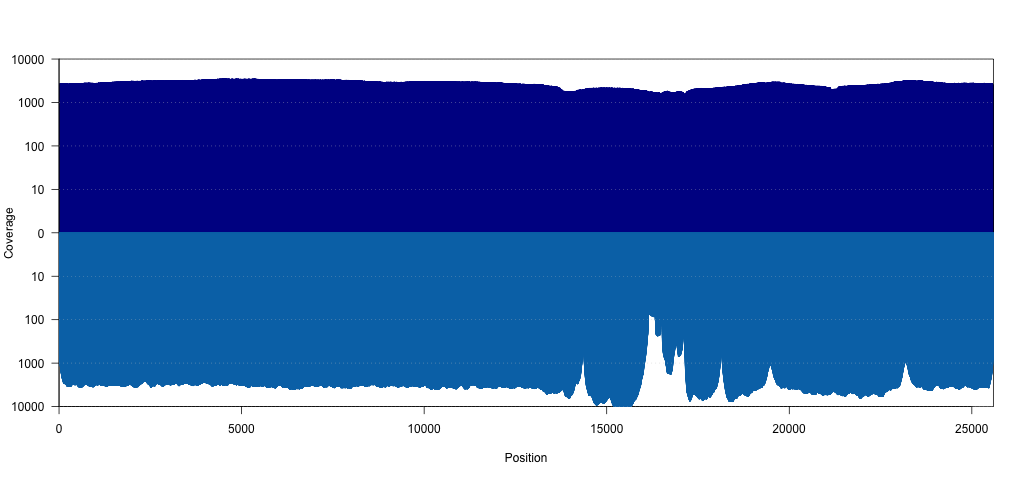


Figure S9. The estimated coverage depths from nanopore (upper dark blue) and Illumina (lower light blue) sequencing reads for *Dynastes* *paschoali* mitogenome.


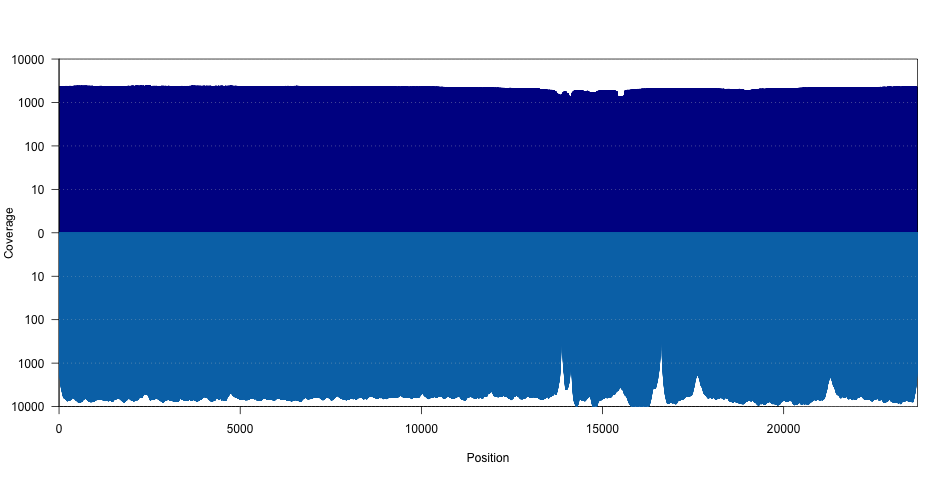


Figure S10. The estimated coverage depths from nanopore (upper dark blue) and Illumina (lower light blue) sequencing reads for *Dynastes* *neptunus* mitogenome.


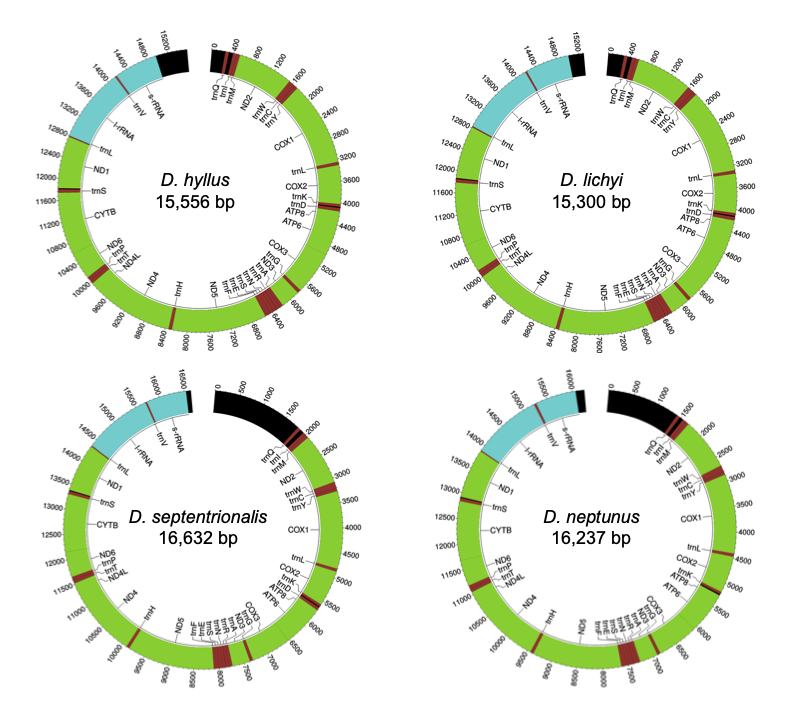


Figure S11. Four example *Dynastes* beetle mitochondrial genomes assembled using only the Illumina sequencing reads. The control region (in black) could not be assembled completely.

Figure S12. Scatter plot between the length of the alignments and the number of parsimoniously informative sites. Red dots indicate results from ribosomal rRNA loci (rrnS and rrnL; or 12S and 16S). In general, the longer the sequence alignments, the more the number of parsimoniously informative sites. Protein-coding loci seem to have a higher ratio of sequence variation than do ribosomal-rRNA loci in the mitochondrial genome.

Figure S13. Scatter plot between the number of parsimoniously informative sites and the calculated *P* values for statistically rejecting the topology of the nuclear-genome tree. Open circles: AU test results; asterisks: SH test results. The horizontal red solid line indicates a *P* value of 0.05. Results from the most frequently used mitochondrial locus for phylogenetics, *COX1*, are highlighted in red.


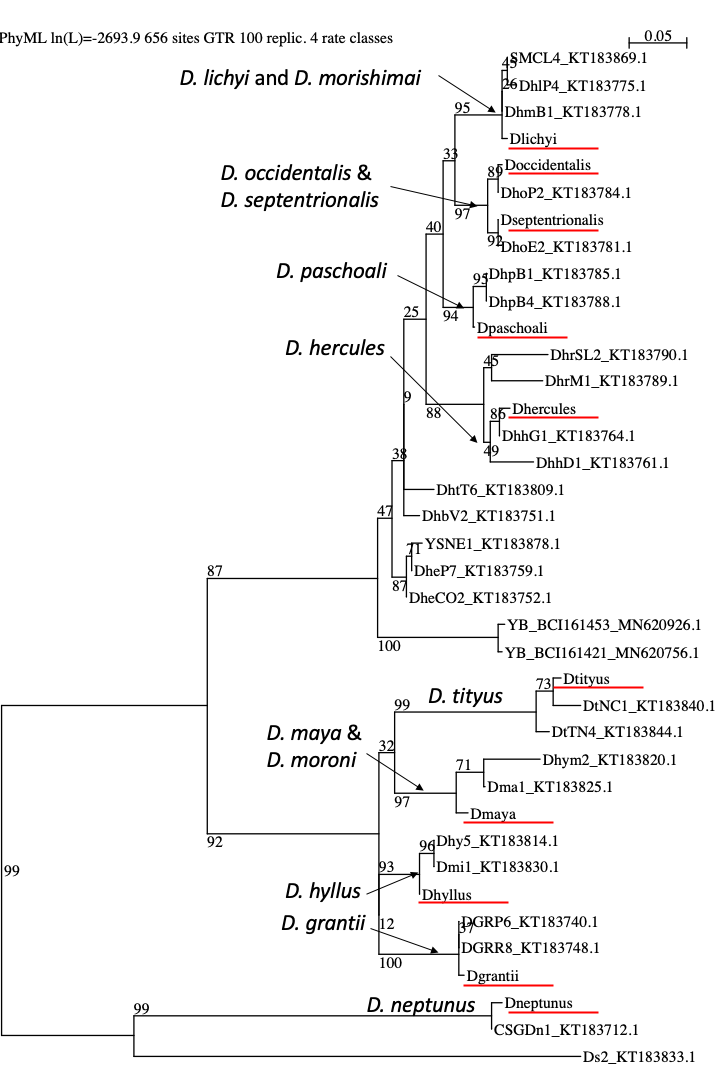


Figure S14. A maximum-likelihood tree based on the mitochondrial *COX1* barcoding region using sequences from the newly assembled mitochondrial genomes and some retrieved sequences published in Huang and Knowles (2016). The newly sequenced data are highlighted with red underlines. Nodal supports were evaluated with 100 bootstrap replicates.

Figure S15. Scatter plot between the calculated dn/ds ratio and the calculated *P* values for statistically rejecting the topology of the nuclear-genome tree (based on AU test).
